# Interactions of Dnd proteins involved in bacterial DNA phosphorothioate modification

**DOI:** 10.3389/fmicb.2015.01139

**Published:** 2015-10-20

**Authors:** Wei Xiong, Gong Zhao, Hao Yu, Xinyi He

**Affiliations:** State Key Laboratory of Microbial Metabolism and School of Life Science and Biotechnology, Shanghai Jiaotong UniversityShanghai, China

**Keywords:** microbial DNA phosphorothioation, DNA enzyme purification, protein interaction technology

## Abstract

DNA phosphorothioation (PT) is the first discovered physiological DNA backbone modification, in which a non-bridging oxygen atom of the phosphodiester bond is replaced with a sulfur atom in Rp (rectus for plane) configuration. PT modification is governed by a highly conserved gene cluster *dndA/iscS-dndBCDE* that is widespread across bacterial and archaeal species. However, little is known about how these proteins coordinately react with each other to perform oxygen–sulfur swap. We here demonstrated that IscS, DndC, DndD and DndE form a protein complex of which the molecular ratio for four proteins in the complex is approximate 1:1:1:1. DndB here displayed little or weak affinity to the complex and the constructs harboring *dndACDE* can confer the host *in vivo* PT modification. Using co-purification and pull down strategy, we demonstrated that the four proteins assemble into a pipeline in collinear to its gene organization, namely, IscS binding to DndC, DndC binding to DndD, and DndD binding to DndE. Moreover, weak interactions between DndE and IscS, DndE and DndC were also identified.

## Introduction

Phosphorothioated nucleotides become more resistant to *in vivo* nuclease decay and survive longer time in the process of targeted gene therapy of some human diseases (Eckstein and Gish, 1989). Prior to its application as a therapeutic tool, DNA phosphorothioation had been uncovered as a physiological modification in which the non-bridging oxygen of the DNA phophodiester bond is replaced with a sulfur atom. This DNA backbone phosphorothioate modification occurred in a DNA sequence-specific and in Rp (rectus for plane) configuration across many bacterial and archaeal species (Wang et al., 2007). Phosphorothioate linkages are susceptible to Tris peroxide, which accumulates on the anode during common or pulsed-field gel electrophoresis, resulting in DNA degradation (Dnd) phenotype (Dyson and Evans, 1998; Zhou et al., 2005). The Dnd phenotype can be suppressed by replacing Tris with Hepes in the electrophoresis buffer or by addition of thiourea (Ray et al., 1992, 1995). Previous studies have shown that phosphorothioate modification is governed by the products of the five clustered *dndABCDE* genes (Zhou et al., 2005). DndA is highly identical to a L-cysteine desulfurase IscS, the major iron-sulfur cluster assembly protein in *Escherichia coli*. Actually in many bacteria, *dnd* gene clusters consist of only *dndBCDE* without a cognate *dndA* (He et al., 2007), and are capable of conferring host *in vivo* DNA phosphorothioation. Gene deletion and complementation in these strains demonstrated that IscS is the sole cysteine desulfurase taking the role for DndA in the sulfur modification (An et al., 2012). *dndB* encodes a repressor that regulates expression of *dnd* gene cluster and deletion of *dndB* does not abolish PT modification (Xu et al., 2009). DndC possesses ATP pyrophosphatase activity (You et al., 2007) and shows sequence similarity to ThiI, which can accept the thiol group (-SH) from IscS in 4-thiouridine modification in tRNA (Kambampati and Lauhon, 2000). DndD known as SpfD in *Pseudomonas fluorescens* Pf0-1, has ATPase activity possibly related to DNA structure alteration or nicking during sulfur incorporation (Yao et al., 2009). Structural analysis of DndE suggested that it is involved in binding nicked dsDNA (Hu et al., 2012), however, mutagenesis of the important amino acids implicated in DNA binding does not abolish phosphorothioate modification (Lai et al., 2014).

Previously, *in vivo* interaction of IscS to respective DndC, D, and E was studied using bacterial two-hybrid system (An et al., 2012). Beyond this, little is known about how Dnd proteins coordinately interact with each other to accomplish PT modification. This study aimed to investigate interaction network among these conservatively oriented Dnd proteins. By using co-expression and co-purification methods, we found IscS, DndC, DndD, and DndE form a large protein complex. The complex was purified to almost homogeneity in this study, and provides solid foundation to future crystallographic analysis of Dnd complex.

## Materials and Methods

IPTG (isopropyl β-D-thiogalactopyranoside), sodium chloride, imidazole, Tris base, were obtained from SangonBiotech, Co. Ltd., (Shanghai).

### Construction of the Relevant *dnd* Gene Over-Expression Vectors

A 5066 bp fragment containing the *dndBCDE* gene cluster (from *dndB* gene start codon to *dndE* end) was amplified by using genomic DNA of *Salmonella enterica* serovar Cerro 87 as template, and inserted behind T7 promoter in pET-28a(+), generating plasmid pDndBCDE_H_ (or pDnd^+^, “H” refers to His-tag). Similar construction strategy was used to generate plasmid pDnd_H_BCDE. Plasmid pDndBCD_H_ was obtained by in-frame deletion of *dndE* (351 bp) from plasmid pDnd^+^, generating DndD fused with His_6_-tag. Plasmid pDndBCE_H_ was obtained from in-frame deletion of *dndD* (1958 bp) from plasmid pDnd^+^. Plasmid pDndDE_H_ was obtained from in-frame deletion of *dndBC* from plasmid pDnd^+^. Plasmid pDndBC_H_ was obtained by in-frame deletion of *dndDE* (2352 bp) from plasmid pDnd^+^. Plasmid pDndE_H_ was obtained from the amplification of a 351 bp *dndE* gene fragment from total DNA of *S. enterica serovar* Cerro 87 and cloning into vector pET-28a(+). Primers used are listed in Supplementary Table S1.

### Growth Conditions, Strains, and Plasmids

The strains and plasmids used for this study are listed in Supplementary Table S2. Luria broth (LB) contains (per liter) 10 g Tryptone, 5 g yeast extract, and 10 g of sodium chloride. *S. enterica serovar* Cerro 87 (wild type strain carrying the *dnd* gene cluster) was cultured in LB medium at 37°C. Isolated total DNA and plasmids were generally stored in autoclaved TE buffer before use. Plasmids were transformed into *E. coli* strain DH10B for construction or BL21 (DE3) for protein expression. All over-expression strains were cultured to about OD_600_ = 0.8, and then induced by using 400 μM IPTG for 20 h at 16°C.

### Purification of Dnd Complex

All experiments were performed at 4°C. Cells were collected by centrifugation at 5000 × *g* for 10 min. Cell pellets were resuspended in lysis buffer (50 mM Tris-HCl pH 7.4, 150 mM NaCl, and 40 mM imidazole) and sonicated. The insoluble fraction was removed by centrifugation at 12,000 rpm for 60 min. The supernatant was loaded onto a Ni–NTA resin (GE) column that had been equilibrated with lysis buffer. After all the extract flowed through, the column was washed with five bed volume of lysis buffer, after which protein complex was eluted with 2.5 bed volume of elution buffer (50 mM Tris-HCl, pH 7.4, 150 mM NaCl, and 500 mM imidazole).

The Heparin affinity and size exclusion chromatography were performed on ÄKTA fast protein liquid chromatography system (GE). The collected eluate was then applied to a HiTrap^TM^ Heparin HP Column, 1 ml (GE) equilibrated with buffer A (50 mM Tris-HCl, pH 7.4, 50 mM NaCl). The complex was then eluted with a linear gradient of buffer B (50 mM Tris-HCl, pH 7.4, 1 M NaCl). The Dnd proteins eluted at 400 mM NaCl. The peak fractions containing the complex were collected and concentrated to 2 mL by using centrifugal filter [Centricon Plus-20 (PL-10), Millipore]. The sample was then applied to Superdex 200 10/30 column (GE) which had been equilibrated with buffer C (50 mM Tris-HCl, pH 7.4, 150 mM NaCl). The same procedure was applied to purify other Dnd proteins. Purified protein was quantified by using Bradford Assay (Thermo Scientific) and stored at -80°C.

### Gel Shift Assay for the Analysis of Dnd Proteins

The purified DndCDE complex (50 μg) was mixed with different amounts of IscS protein (8, 16, 24, 32 μg) at room temperature for 10 min in buffer C supplemented with 10% glycerol. Then about 20 μg protein samples were loaded directly to gradient native PAGE gel and the electrophoresis was performed at 4°C for 12 h with constant current set at 5 mA. The gel was then stained with Coomassie Blue for detecting.

### Densitometry Analysis of Coomassie Brilliant Blue-Stained SDS-PAGE Protein Gel and Determination of the Molar Ratio among Subunits of Dnd Complex

The concentrations of proteins were set about 0.5 mg/ml, and then were separated by 15% SDS-PAGE. Gel was stained by coomassie brilliant blue R250 and photographed by using Bio-Rad Molecular Imager system. Protein bands in each lane was scanned and analyzed by Bio-Rad Image Lab Software. The staining intensity of each protein band was quantified. The molar ratio among subunits of the complex was calculated according to the staining intensity and the molecular weight of each protein.

## Results

### Co-purification Experiments show that Dnd Proteins Form a Complex

**FIGURE 1:**
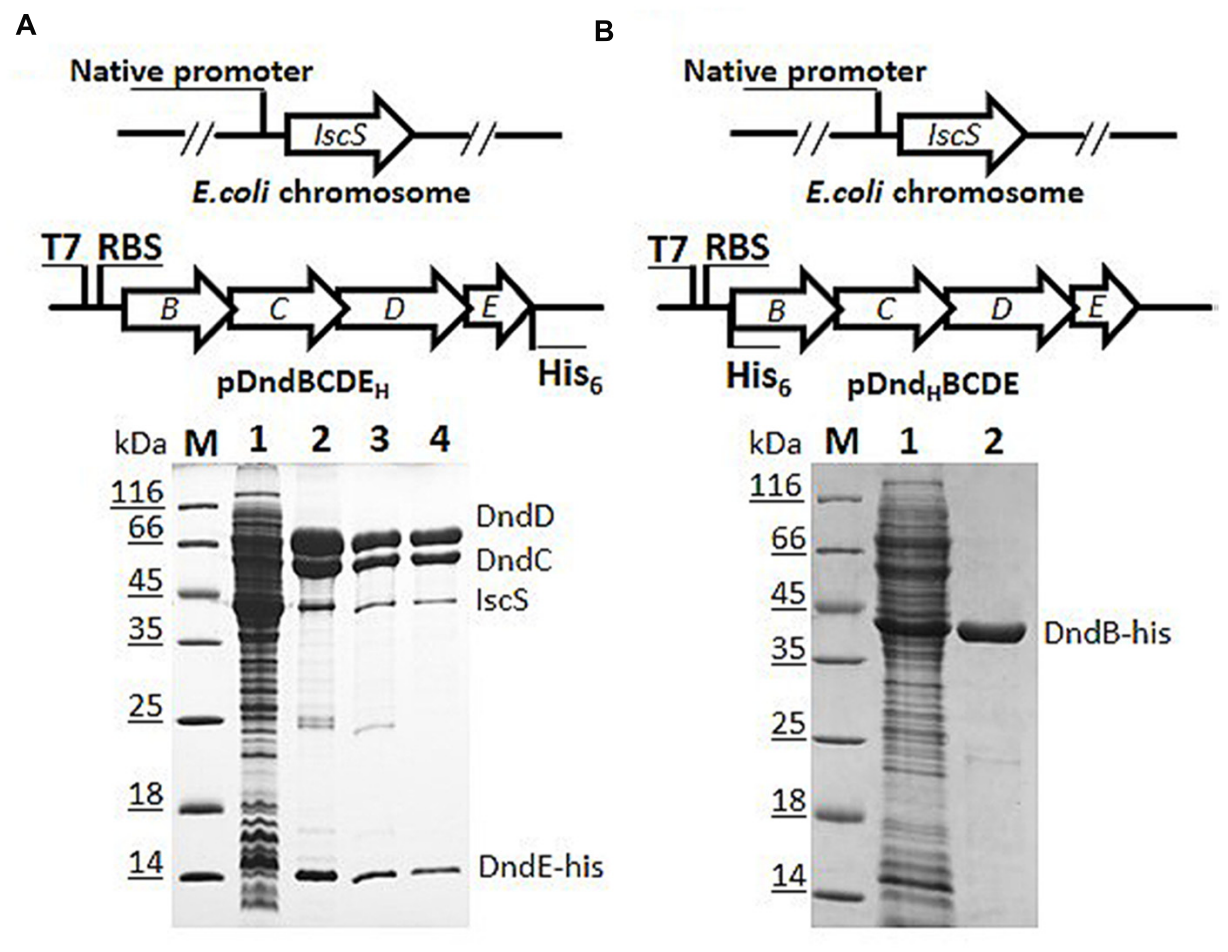
**Constructs for co-expression and purification of Dnd proteins with his-tag fused either to DndB or to DndE on pET28a (+). (A)** His-tagged DndE can purify Dnd protein by nickel affinity (lane 2), heparin affinity (lane 3), and size exclusion chromatography (lane 4). Slot 1 is the supernatant for the host expressing pDndBCDE_H_, H here represents the his-tag. The eluted protein was resolved by 15% SDS-PAGE. The identities of the proteins were confirmed by mass spectrometry and the weak protein band below DndC protein is IscS from the *Escherichia coli* BL21 (DE3) host rather than DndB. M: protein markers. **(B)** His-tagged DndB cannot co-purify other Dnd proteins.

In the process of overexpression and purification of individual Dnd protein in *E. coli* BL21 (DE3), DndC and DndD from *Salmonella* were always in the fraction of pellet even after tremendous conditions optimization, and hinder the enzymatic study of Dnd proteins. We therefore chose to express the whole *dndBCDE* from *Salmonella* as the four genes formed an operon (Xu et al., 2009) and is functional in DNA phosphorothioate modification together with a standalone IscS gene (**Figure 1A**). To facilitate the purification of the complex, a 6xHis tag was either used to displace the stop codon of *dndE* or inserted at the start codon of *dndB* (**Figure 1**).

**FIGURE 2:**
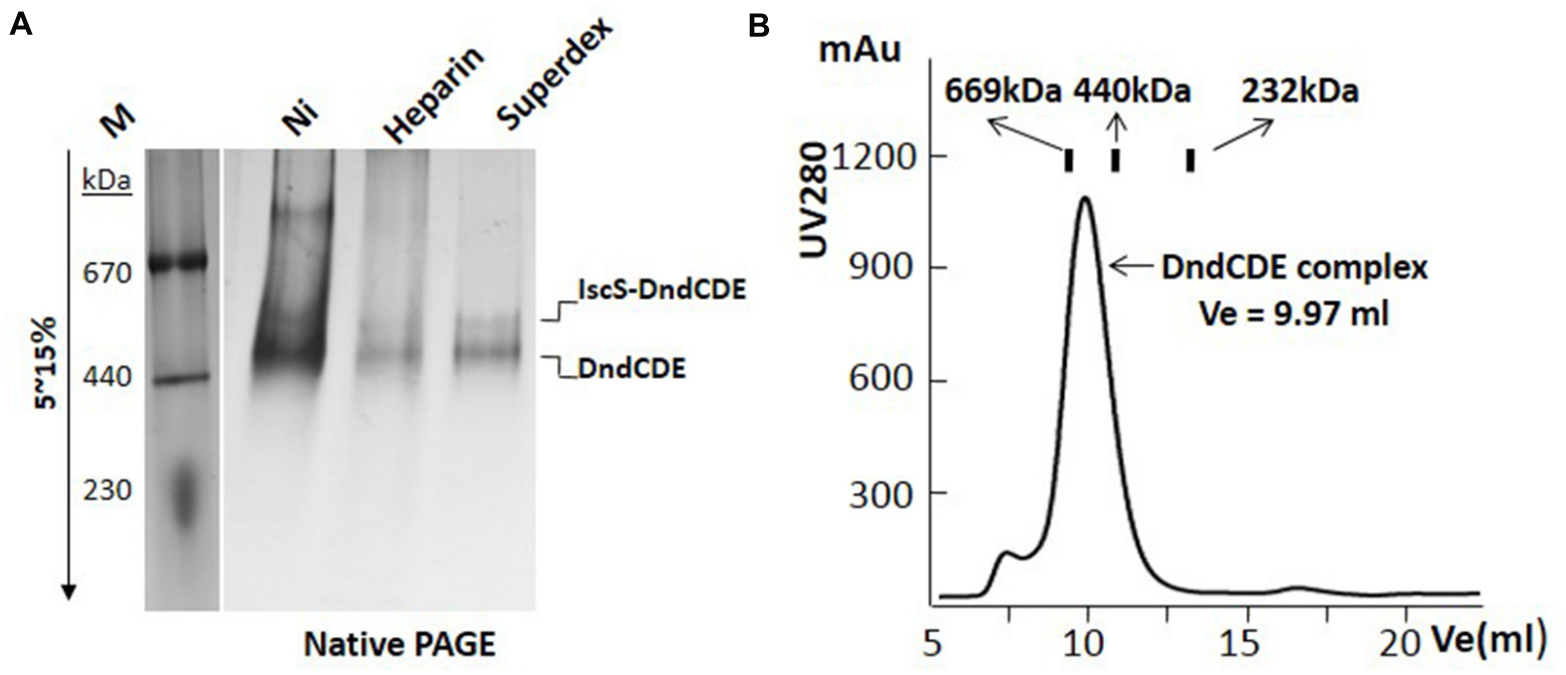
**DndCDE complex is determined by gradient native-PAGE and gel filtration analysis. (A)** The integrity of the Dnd protein complex was analyzed by 5–15% gradient native PAGE after each purification step. M: protein markers; Ni: Nickel affinity; Superdex: superdex 200. **(B)** The molecular weight of the purified Dnd complex is calibrated by size exclusion chromatography. Thyroglobulin (669 kDa), ferritin (440 kDa), catalase (232 kDa) were loaded into the superdex 200 10/300 GL column respectively. The Dnd complex elutes at the position corresponding to the size around 600 kDa.

After IPTG induction, protein bands in the supernatant corresponding to the size of DndB, C, D, E were visibly much more thicker in the SDS-PAGE (Slot 1 in **Figures 1A,B**). After nickel affinity purification, at least six bands were co-purified from the soluble fraction for the construct with 6xHis tagged to C-terminus of DndE (Slot 2, **Figure 1A**). These proteins were further purified by Heparin affinity and size exclusion chromatography (Slots 3 and 4, **Figure 1A**), and each band was excised from the gel and subjected to mass spectroscopy analysis. The final four protein bands on the SDS-PAGE gel (Slot 4, **Figure 1A**) were revealed to be IscS, DndC, DndD, and DndE-His. It is particularly worthy of being mentioned that the standalone, constitutively expressed IscS on the chromosome was co-purified by this strategy. In sharp comparison, only one band corresponding to DndB was purified when the 6xHis was tagged to the N-terminus of DndB (**Figure 1B**), indicating that DndB is not a component of the DndCDE complex. For the construct with DndE his-tagged, the eluted protein after each purification method was individually checked by gradient native PAGE (**Figure 2A**), one minor band of bigger size and one major band of smaller size were detected, and respectively proposed as DndCDE complex and IscS-DndCDE complex based on the protein identities determined by LC/MS analysis (Shanghai Sangon Biotech, Co. Ltd.), the approximate size for the major DndCDE is ca. 460 kDa in the native PAGE but is ca. 600 kDa in the size exclusion chromatography as compared to standards (**Figure 2B**, Supplementary Table S3). These observations together demonstrated that IscS, DndC, D, and E but DndB form and sustain a complex that survived multiple purification steps (**Figure 1A**).

### Each of Dnd Protein in the Complex is in Equal Molar Ratio

**FIGURE 3:**
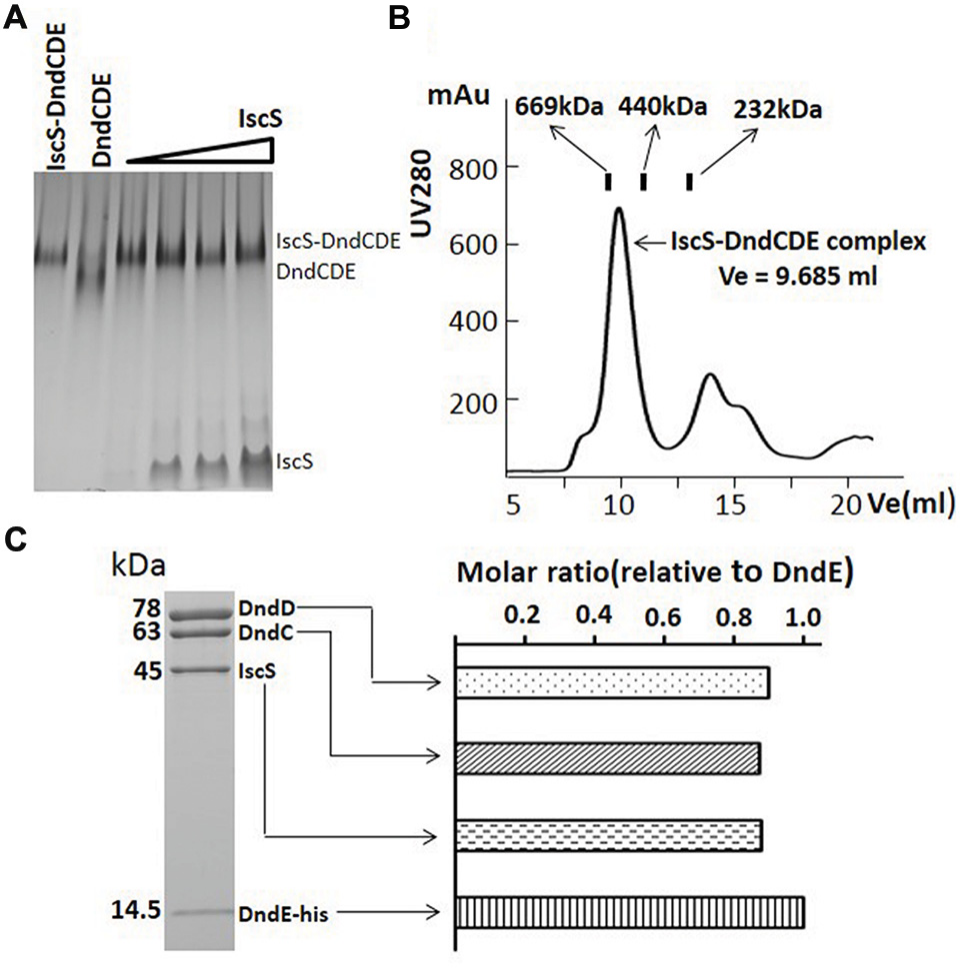
**IscS binds to DndCDE complex to form IscS-DndCDE complex. (A)** DndCDE complex saturated with increasing iscS were analyzed by 5–15% gradient native PAGE. **(B)** Gel filtration analysis of putative IscS-DndCDE complex. The column used are the superdex 200 and 10/300 GL. The digits are same as that in legend for **Figure 2B**. **(C)** SDS-PAGE gel analysis of the complex eluted after Superdex purification. The subunit constitution of the complex, the molecular weights of proteins are labeled on the left side of the gel. The comparative abundance of each of Dnd protein calculated based on densitometry scanning of the protein bands on Coomassie brilliant blue-stained SDS-PAGE gels.

Since the intensity of IscS on the SDS-PAGE gel is obviously weaker than anyone of DndC, D or E, we attempted to know if the lower concentration of IscS in the complex was caused by the unavailability of IscS that is constitutively expressed on the chromosome or by its weak affinity to other Dnd proteins. IscS was therefore over-expressed in *E. coli* and purified. The DndCDE complex was then mixed with increasing amount of IscS and separated on the gradient native gel. One band higher than that for DndCDE formed and kept the same size with increasing IscS concentration (**Figure 3A**). The size of the new complex is approximately 670 kDa determined by size exclusion analysis as compared to the protein standards (**Figure 3B**, Supplementary Table S3). The complex saturated by IscS was purified by superdex 200 exclusion and checked by SDS-PAGE (**Figure 3C**) to see molar ratio among the four proteins. Densitometry analysis of each band in the SDS-PAGE protein gel revealed that the molar ratio of IscS: DndC: DndD: DndE is approximate 1: 1: 1: 1 (**Figure 3C**).

### Interaction Network among the Subunits of the IscS-DndCDE Complex

**FIGURE 4:**
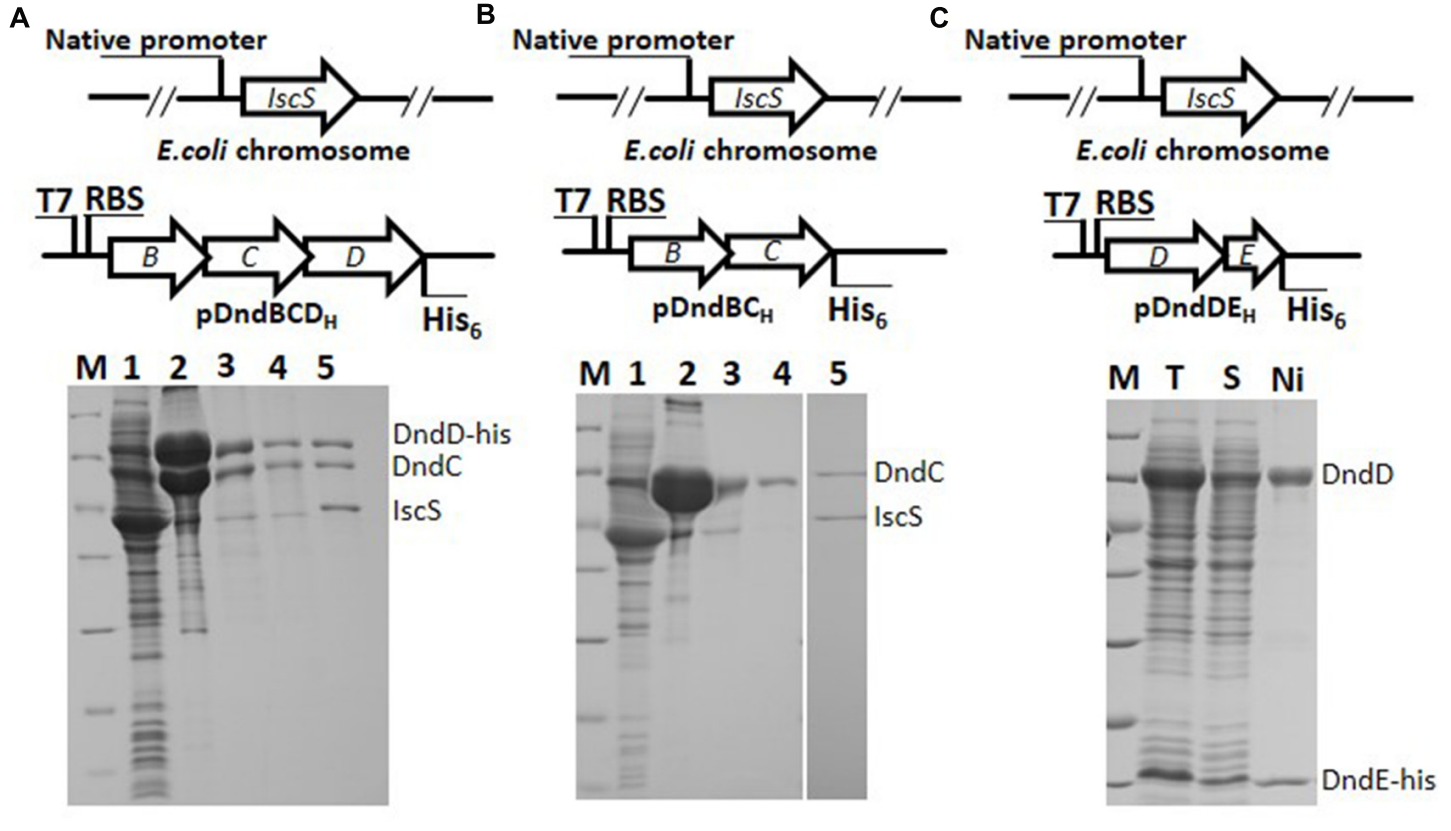
**The evidence for the strong affinity between DndC&D, IscS&DndC DndD&DndE by copurification. (A)** His-tagged DndD can pull down DndC. Lane 1: supernatant of lysate of *E. coli* BL21(DE3) expressing *dndBCD* with D his-tagged; lane 2: purified by nickel affinity column; lane 3: purified after heparin affinity; lane 4: purified by superdex 200 10/300 GL column; lane 5: IscS-DndCD complex separated by superdex 200 10/300 GL column. Elutes are resolved by 15% SDS-PAGE. **(B)** His-tagged DndC can pull down the chromosomally expressed IscS. Lane 1: supernatant of lysate of *E. coli* BL21(DE3) expressing *dndBC* with C his-tagged; lane 2: purified after Ni affinity; lane 3: purified by heparin affinity column; lane 4: purified after superdex 200 10/300 GL column; lane 5: strep-tagged IscS was mixed with DndC purified by nickel and heparin affinity chromatography, then loaded on size exclusion chromatography superdex 200 10/300 GL column. Elutes are resolved by 15% SDS-PAGE. **(C)** His-tagged DndE pull down DndD. The *dndDE*_H_ construct was over-expressed. Proteins after nickel purification were analyzed by 15% SDS-PAGE. Protein bands correspond to DndD and DndE were indicated.

**FIGURE 5:**
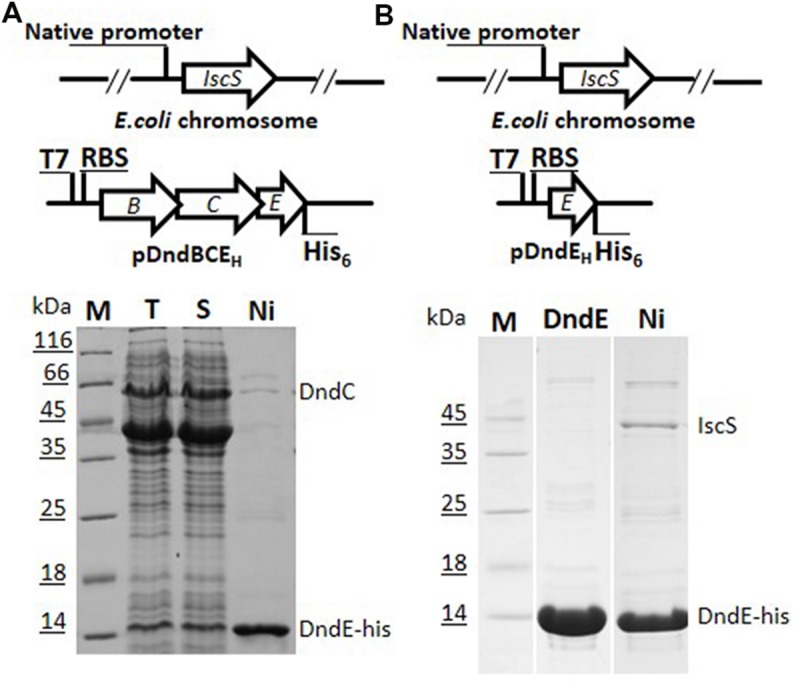
**Weak affinity for DndE to DndC and IscS. (A)** DndC interacts with DndE. After nickel affinity purification, proteins were separated by SDS-PAGE. The identity of DndC was confirmed by mass spectrometry. **(B)** IscS interacts with DndE. Lysate for construct over-expressing DndE-his_6_ was mixed with that for construct over-expressing IscS-strep tag and purified by nickel affinity column.

In order to know interactions among Dnd proteins, co-expression, and co-purification strategy was employed. To study interaction between DndC and DndD, *dndE* was in-frame deleted and a His_6_ tag was fused at the C-teminus of DndD protein. After induction and purification by nickel affinity chromatography, nearly equal molar of DndC to DndD was co-purified, they kept at equal concentration in the Heparin affinity and size-exclusion chromatograpgy, indicating that DndC and DndD bind directly to each other (**Figure 4A**). The molar ratio of DndC and D was quantified by measuring the staining intensity of protein bands as described in the materials and method. Purified IscS could also bind directly to complex of DndCD (Slot 5, **Figure 4A**), however, which one of them is associated with IscS is unknown. To address this question, we co-expressed DndBC as DndC alone is insoluble, and found that His-tagged DndC can pull down the chromosomally expressed IscS (Slots 2 and 3 in **Figure 4B**), but IscS fell off from the complex after size exclusion (Slot 4 in **Figure 4B**), demonstrating that IscS binds directly to DndC, but the affinity between them is weaker than that between DndC and DndD. SDS-PAGE analysis revealed that equal molar of IscS and DndC can form a IscS-DndC complex when adding purified Strep-IscS to DndC that is bound to nickel resin (Slot 5, **Figure 4B**). As DndD is insoluble if expressed alone, we then co-expressed DndDE-6xHis, and found DndD and DndE form a complex in a molar ratio of 1:1, demonstrating that DndD binds directly to DndE. However, this complex precipitated within an hour. For this construct, no IscS can be pulled down implying that both DndD and DndE has weak or little affinity to IscS. In order to analyze the interaction between DndC and DndE, *dndD* was deleted and *dndBCE* were co-expressed, nickel affinity purification result in co-purification of trace of DndC, implying that DndC binds to DndE with very weak affinity (**Figure 5A**). DndE-His also cannot pull down the chromosomally expressed IscS, but when adding Strep-IscS into DndE bound to nickel affinity column, the eluted fraction contains very low amount of IscS, demonstrating that DndE has weak affinity to IscS (**Figure 5B**).

### IscS-DndBCDE and IscS-DndCDE are Sufficient to Confer the Host Phosphorothioate Modification

**FIGURE 6:**
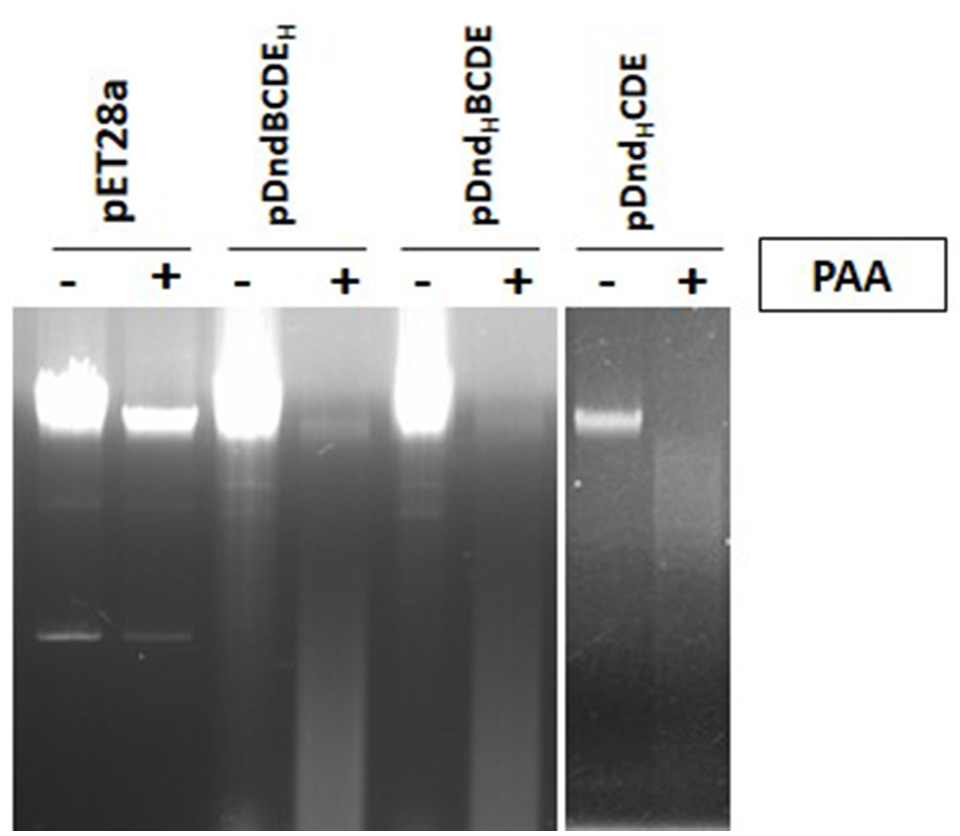
**Dnd phenotype assay of total DNA from *E.coli* BL21 (DE3) expressing the *dnd* genes.** PAA is abbreviated for peracetic acid, ‘+’ indicates PAA treatment, ‘-’ indicates no PAA treatment. DNA samples were separated in 0.7% agarose gel supplemented with 100 mM thiourea to Tris-acetate electrophoresis buffer. DNA smear indicates DNA PT modification. pET28a: the total DNA of *E. coli* BL21 (DE3) harboring pET28a as the control; pDndBCDE_H_, pDnd_H_BCDE and pDnd_H_CDE represents the total DNA of *E. coli* BL21 (DE3) expressing these three constructs respectively. H represents his-tag. Each line above ‘+’, ‘-’ corresponds one plasmid or one construct.

As a 6xHis tag was fused to either DndB or DndE for above protein purification, we wonder if *E. coli* BL21 (DE3) expressing these engineered protein can perform DNA PT modification. Dnd phenotype that is characteristic of DNA phosphorothioate modification for these strains are measured as described in (Xie et al., 2012). Both constructs can confer host PT modification, demonstrating that His-tag doesn’t affect PT modification (**Figure 6**, left panel). As only four Dnd proteins formed complex revealed by this study, *dndB* was deleted and a 6xHis was tagged to N-terminus of DndC to study the DNA PT modification. As expected, the Dnd phenotype was observed during electrophoresis (**Figure 6**, right panel), demonstrating overexpression of IscS and his tagged DndCDE proteins were fully functional in the host.

## Discussion

### Binding Affinity between IscS/DndA and Other Four Dnd Proteins

As the major iron sulfur assembling protein, three independent interfaces of IscS that interacts with proteins from different sulfur processing pathways were revealed (Shi et al., 2010). Among these downstream proteins, ThiI, which accepts the thiol group (-SH) from IscS, a homolog to DndC, interacts directly with IscS in tRNA 4-thiouridine modification. In this study, we provide direct evidence that chromosomally expressed IscS could be co-purified by his-tagged DndC (**Figure 4B**). This observation is consistent to the result of interactions between IscS and ThiI (Kambampati and Lauhon, 2000) as well as that of bacterial two-hybrid system (An et al., 2012). The interface between IscS and ThiI might be employed in the binding between IscS and DndC. In addition, much weaker binding affinity of IscS to DndE was also observed using the pull down strategy. By comparison, no visible IscS can be co-purified by expressed complex DndDE (**Figure 4C**), implying that expressed DndD has little affinity to IscS.

### Interaction between DndC and Other Dnd Proteins

In addition to binding to DndA/IscS, DndC exhibit strong affinity with DndD (**Figure 4A**), indicating that DndC might plays a role of ‘connector’ in accepting sulfur from DndA/IscS and transferring it to DndD as there is no direct interaction between IscS and DndD. Moreover, beyond the regulation of the *dndBCDE* operon, DndB might enhance the solubility and/or stability of DndC as DndC often incorrectly folds in the inclusion bodies when expressed alone but is soluble when DndB is co-expressed (**Figure 4B**). DndC also displayed comparatively weak interaction to DndE as His-tagged DndE can catch low amount of DndC during the nickel affinity purification.

### Interaction of DndD with Other Dnd Proteins

DndD was identified to possess ATPase activity and shows homology to protein that is necessary for structure maintenance of chromosome (SMC). DndD is thus proposed to be involved in sulfur incorporation into the DNA backbone. Moreover, DndD also has strong affinity to DndE, implying that it contains at least two different interfaces in the Dnd complex. Like DndC, DndD from *Salmonlla* alone often incorrectly folds into the inclusion bodies, but when co-expressed with DndE, DndDE complex can be formed and purified, but this sub-complex lasts at most for 1 h in the buffer and precipitates. Interaction between DndD and DndC sealed this interface that might be hydrophobic and thus stabilize the complex in the solution. When apart, the hydrophobic interface for either of DndD or DndC exposed to water and both of them become insoluble. DndD becomes temporally stable upon the binding to DndE by using an interface other than to DndC. However, DndE is a relatively small protein of 13.5 KD, and stabilization of DndD by DndE might be impaired when other putative hydrophobic interface (s) on DndD exposed to the water.

### The Stoichiometry for Dnd Complex

Adding purified IscS to the sub-complex of DndCDE obviously formed a bigger complex (**Figure 3B**), which is purified to the homogeneity with heparin affinity and size exclusion method. The molecular ratio of each component in this purified complex was calculated as approximate 1:1:1:1 by three separately purified complex. However, it is very hard to predict the molecular weight of this complex as the shape of this complex is unknown. In native PAGE gel, the size of the complex is ca. 500 kDa (**Figure 2A**), but in the superdex 200 filtration assay, its size is close to 669 kDa as compared to varied protein standard (Supplementary Table S3). The accurate stoichiometry for this complex can be elaborated by the protein crystallographic analysis. The complex of essential IscS/DndA-DndCDE purified by this work provides strong foundation to this aim and finally to uncover the detail of the oxygen–sulfur swap.

## Conflict of Interest Statement

The authors declare that the research was conducted in the absence of any commercial or financial relationships that could be construed as a potential conflict of interest.
