# Supplementary material for: Interactions of Dnd proteins involved in bacterial DNA phosphorothioate modification
Source: Front Microbiol. 2015 Oct 20;6:1139. doi: 10.3389/fmicb.2015.01139 (PMC4611135; doi:10.3389/fmicb.2015.01139)
Supplement: Supplementary file 1 [file Data_Sheet_1.DOC]

**Supplementary material**

### **Interactions of Dnd Proteins involved in bacterial DNA phosphorothioate modification**

Wei Xiong^1^, Gong Zhao, Hao Yu and Xinyi He*^1^

^1^State Key Laboratory of Microbial Metabolism and School of Life Science & Biotechnology, Shanghai Jiaotong University, Shanghai 200030, China

*Corresbonding author

Email: [xyhe@sjtu.edu.cn](mailto:xyhe@sjtu.edu.cn)

Tel: 021-62932943; Fax: 021-62932418

**Table S1 primers**

| **PRIMERS** | **SEQUENCE**  **(underline indicates restriction site)** | **USE** |
| --- | --- | --- |
| pDndBCDE_H-_UP | CTTGCCATGGCTAGTGTTGATGCAGA | Amplify *dnd* genes |
| pDndBCDE_H-_DP | CGGCTCGAGTATATTCTTTGAAAAATCTT |
| pDnd_H_BCDE-UP | GGAATTCCATATGGCTAGTGTTGATGCAGA | Amplify *dnd* genes |
| pDnd_H_BCDE-DP | CGGCTCGAGTCAGACAGCCTCCTTGGTTAATT |
| pDndBCD_H_-UP | CACCACCACCACCACCACTGA | Deletion of *dndE* |
| pDndBCD_H_-DP | GACAGCCTCCTTGGTTAATT |
| pDndBCE_H_-UP | ACTCATGAGGCAACCATTTG | In-frame deletion of *dndD* |
| pDndBCE_H_-DP | ATGCGGGCGAATCGTCTGTG |
| pDndDE_H_-UP | ATGCGGGCGAATCGTCTGTG | In-frame deletion of *dndBC* |
| pDndDE_H_-DP | CATGGTATATCTCCTTCTTA |
| pDndBC_H_-UP | CACCACCACCACCACCACTGA | Deletion of *dndDE* |
| pDndBC_H_-DP | TGAAACCTGGCTATTTCTGG |
| pDndE_H_-UP | CTTGCCATGGGCCTCCCGAATCGAATGGT | Construction of pDndE_H_ |
| pDndE_H_-DP | CGGCTCGAGTATATTCTTTGAAAAATCTT |
| pIscS- UP | CATGCCATGGCAAGCTGGAGCCACCCGCAGTTCGAAAAGATGAAATTACCGATTTATCTCG | Construction of pIscS |
| pIscS- DP | CCGGAATTCTTAATGATGAGCCCATTCGA |

**Table S2 Strains and plasmids**

| **STRAINS** | **CHARACTERISTICS** | **REFERENCE** |
| --- | --- | --- |
| *Salmonella enterica serovar* Cerro 87 | Source of the *dndBCDE* genes, Dnd phenotype positive |  |
| *E. coli* DH10B | Non-restricting host strain for gene cloning | Novagen |
| *E.coli* BL21 (DE3) | Host for heterologous expression | Novagen |
| **PLASMIDS** |  |  |
| pET28a | *E. coli* expression vector, Kan^r^ | Novagen |
| pDndBCDE_H_(pDnd+) | *dndBCDE* (four genes co-transcription) in pET28a. His_6_-tag with DndE, Dnd phenotype positive, Kan^r^ | This work |
| pDnd_H_CDE (pDnd+) | *His-tag was inserted at the N-terminus of dndC* in pET28a. Dnd phenotype positive, Kan^r^ | This work |
| pDndBCD_H_ | *dndBCD* (three genes co-transcription) in pET28a. His_6_-tag with DndD, Kan^r^ | This work |
| pDndDE_H_ | *dndDE* (two genes co-transcription) in pET28a. His_6_-tag with DndE, Kan^r^ | This work |
| pDndBCE_H_ | *dndBCE* (three genes co-transcription) in pET28a. His_6_-tag with DndE, Kan^r^ | This work |
| pDndBC_H_ | *dndBC* (two genes co-transcription) in pET28a. His_6_-tag with DndC, Kan^r^ | This work |
| pDndE_H_ | *dndE* in pET28a vector. His_6_-tag with DndE, Kan^r^ | This work |
| pDnd_H_BCDE | *dndBCDE* in pET28a. N-terminal His_6_-tag with DndB*,* Dnd phenotype positive, Kan^r^ | This work |
| pIscS | *E. coli iscS* gene was cloned into pET28a. N-terminal strep tag II, Kan^r^ | This work |

**Table S3** Protein standards to determine the molecular weight of Dnd complex (in red) via gel filtration

| Standard | Elution  time | Elution Volumn  (Ve) | Ve/Vo | MW | lg MW |
| --- | --- | --- | --- | --- | --- |
| Catalase | 25.31 | 12.67 | 1.58 | 232 000 | 5.37 |
| Ferritin | 21.98 | 10.99 | 1.37 | 440 000 | 5.64 |
| Thyroglobulin | 19.19 | 9.60 | 1.20 | 669 000 | 5.83 |
| DndCDE | 19.94 | 9.97 | 1.25 | **601KD** | **5.77** |
| IscS-DndCDE | 19.37 | 9.685 | 1.21 | **664KD** | **5.82** |
| **Blue dextran** | **16.00** | **8.00** | **1** | **2000 000** |  |
